# A Prospective Analysis of Cognitive Functions in Stable Nonhypoxemic Chronic Obstructive Pulmonary Disease Patients at a Tertiary Care Center, Jaipur

**DOI:** 10.31662/jmaj.2024-0398

**Published:** 2026-03-06

**Authors:** Pratima Agrawal, Sudhanshu Kacker, Neha Saboo

**Affiliations:** 1 Department of Physiology, RUHS College of Medical Sciences, Jaipur, Rajasthan, India

**Keywords:** Chronic Obstructive Pulmonary Disease, Cognitive functions, Nonhypoxemic patients, Forced Vital Capacity, Forced expiratory volume in first second

## Abstract

**Introduction:**

Chronic obstructive pulmonary disease (COPD) is a complex, multi-component disorder characterized by a long-lasting, irreversible airflow limitation due to inflammation, which leads to narrowing of the airway lumen. A variety of influencing factors may cause cognitive impairment (CI) in patients with COPD, including age, disease duration, severity, hypercapnia, smoking, and vascular dysfunction. This study assessed cognitive functions in non-hypoxic stable COPD patients.

**Methods:**

An observational study was conducted in the Department of Physiology and Medicine, Rajasthan University of Health Sciences College of Medical Sciences (RUHS CMS). A total of 120 subjects were recruited. The cases (group A) were selected according to the Global Initiative for Obstructive Lung Disease (GOLD) classification. GOLD stage 1 and 2 patients confirmed by spirometry. Controls (group B) were selected as age-matched healthy volunteers(HVs) from the society. The Mini-Mental State Examination (MMSE) and event-related potential (P300) were used to assess cognitive functions in both groups. Data was analyzed using appropriate tests, and p < 0.05 was considered significant.

**Results:**

The mean MMSE score was found to decrease in Group A compared to Group B. The mean MMSE score in group A was 24.65 with an standard deviation of ± 2.05; in group B, it was detected as 27.56 ± 2.15 (p < 0.00001). The mean latency of the P300 wave was prolonged in some cases, and a decrease in the Amplitude of the P300 wave was observed in some cases compared to the control group. Mean latency in case group 298.48 ± 21.98 milliseconds compared to control group 265.89 ± 15.80 milliseconds, with a significant p-value <0.0001. The mean amplitude in group A was detected as 4.25 ± 1.502 μV, compared to a mean of 6.06 ± 1.94 μV in group B.

**Conclusion:**

Patients with COPD have a higher risk of CI compared to the age-matched healthy volunteer group.

## Introduction

Chronic obstructive pulmonary disease (COPD) is a complex, multi-component disorder characterized by a long-lasting, irreversible airflow limitation resulting from chronic airway inflammation, which leads to narrowing of the airway lumen. It is associated with many psychological and social issues, which are frequently related to multiple comorbidities, such as cardiovascular disease, anemia, osteoporosis, and other extra-pulmonary manifestations ^(1)^. According to World Health Organization Global Health Estimates 2019, it is the third leading cause of death worldwide, and in India, after road traffic accidents, it is the second most prevalent cause of death ^(2)^. Age-specific prevalence of COPD increases rapidly after the age of 30 years ^(3)^. COPD has many systemic effects that cause the brain to be susceptible to impaired cognitive functions, and this risk increases with age and disease severity progression. The mental action or intellectual process of acquiring, understanding, and using knowledge or information through senses, experience, and thinking is the main definition of the word ‘cognition’ through which human behavior can be adapted to new situations and changes in preferences ^(4)^. Different cognitive processes are divided into six essential neuropsychological domains: executive functions, language, visuospatial and motor functions, attention/concentration, social cognition/emotions, learning, and memory ^(5)^. The specific functions of each domain determine personal intellectual skills and knowledge by providing them with both basic and more complex capabilities ^(6)^. Global Initiative for Chronic Obstructive Lung Disease (GOLD) (2020) stated that the average prevalence of cognitive impairment (CI) in COPD is 32% ^(7)^. The prevalence of CI in patients with COPD (17-56.7%) is significantly higher compared to healthy controls (12-12.7%). According to various studies, this difference depends on the study population and the method of neuropsychological assessment ^(8)^. A study by Villeneuve et al. ^(9)^ reported that, on average, the incidence of CI in patients with COPD is 36%, compared to individuals in the general population. In contrast, only 12% of individuals are cognitively impaired ^(9)^. Hung et al. ^(10)^ concluded that individuals with severe COPD, defined as those with oxygen dependence or disease-related activity limitations, were found to have lower cognitive performance over 6 years of follow-up. Evidence of CI in non-hypoxemic individuals, together with a consistent pattern of cognitive deficit, suggests that COPD is associated with neuronal damage or dysfunction that is separate from other comorbidities, such as vascular disease. According to a study, even after adjustment of data for smoking habits, sex, education, and age, the estimated prevalence of CI increased in patients with COPD, which indicates that lung dysfunction is a risk factor for CI ^(11)^. As assessed by activities of daily living, the level of functioning is reduced in individuals with cognitive dysfunction ^(12), (13)^. Cognitive dysfunction is associated with poor compliance with medication and oxygen therapy, and this poor compliance increases the risk of acute exacerbation. Many established tools are used for assessments of cognition. These tools are carefully constructed to evaluate neuropsychological domains, including abstract reasoning, language, memory, executive function, visuospatial skills, and attention ^(14), (15)^. Most previous studies have used established mental status screening tools for assessing cognitive dysfunctions, such as the Montreal Cognitive Assessment (MoCA) or Mini-Mental Status Exam (MMSE) ^(16)^. Multiple cognitive domains can be evaluated using mental status screening, a well-researched, efficient, and easy-to-apply modality. Evaluation of cognitive functions, including attention, short-term memory, information processing speed, and P300 testing, can be used as an effective electrophysiological method in patients with vestibular-related dizziness or vertigo. The P300 is a low-cost, non-invasive, and reproducible test of psychological-neurophysiological response with high sensitivity ^(17)^. Patients with COPD have a high prevalence of CI as compared to same-age healthy volunteers, and this CI is associated with many detrimental consequences on the functional status and quality of life of these patients. This suggests that, within the context of respiratory treatment, assessing cognitive functions and addressing CI in these patients is also a necessary step. CI should be detected early to prevent or delay its underlying processes and minimize potential obstacles in the therapeutic strategy ^(18)^. A comprehensive pulmonary rehabilitation program may be beneficial in patients with COPD who exhibit evidence of CI. This study highlighted the importance of finding a correlation between cognitive functions in stable patients with non-hypoxemic COPD. In this study, subjective (MMSE questionnaire) and objective (event-related potential [ERP]) assessments of cognitive functions were done in stable patients with COPD.

## Materials and Methods

**Figure 1.**
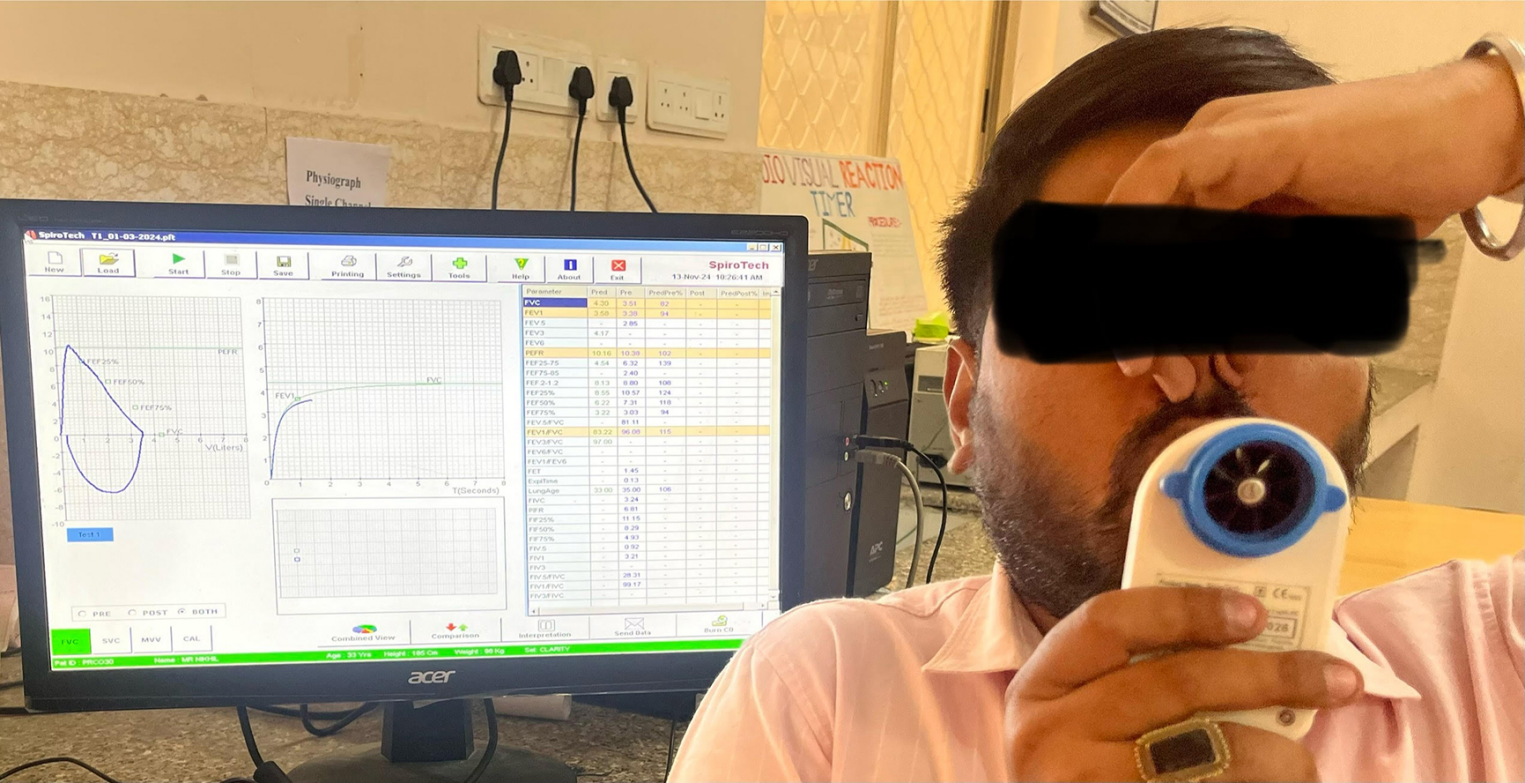
Spirometric evaluation of lung functions.

After obtaining protocol approval from the institutional ethics committee (RUHS College of Medical Sciences, Jaipur) (EC-P-35-23), subjects were recruited for a period of 3 months, from February to April 2024. Recruitment of 120 subjects (60 in group A and 60 in group B) was conducted. The sample size was calculated at a 95% confidence level, an Alpha error of 0.05, and an absolute allowable error of 5%, considering a 10% dropout or non-response rate. Group A was recruited from the medicine outdoor patient department of RUHS-CMS and its associated hospitals in Jaipur. Age-matched healthy volunteers (group B) were taken from society for comparison. The inclusion criteria for recruitment to group A were as follows: age group, 30 to 50 years; GOLD stage 1 and 2 (stages of COPD according to GOLD); and SpO_2_ of patients >90% to rule out any hypoxemia. All patients who had a pulmonary disease other than COPD, cardiovascular disease, hypertension, diabetes, thyroid dysfunctions, systemic disorders, psychological conditions, auditory dysfunctions, and a history of drug abuse or alcohol were excluded from our study. All subjects of both groups verbally explained the purpose of the study, and a participant information sheet was provided to them. After explaining the procedure and its purpose, written informed consent was obtained from the subjects who were willing to participate. Socio-demographic data, including age, sex, menstrual history of female participants, residence, history of migration, religion, education, occupation, and income per capita, and smoking history in packs-year, were recorded. Socioeconomic classes were calculated according to the modified Kuppuswamy scale. Anthropometric data (height in meters, weight in kg, body mass index (BMI) according to weight/ height^2). Environmental history, passive smoking, type of house, kitchen type, cooking duration, family history of COPD, childhood respiratory infections, work exposure to dust, and chemicals were taken. Vital parameters (pulse, blood pressure, respiratory rate, oxygen saturation), spirometric indices forced vital capacity (FVC), forced expiratory volume in 1 second (FEV1), ratio of FEV1 and FVC, and cognitive parameters, MMSE score, latency (ms), and amplitude (μV) of event-related potential (ERP/P-300) were recorded. Blood pressure was checked by a digital sphygmomanometer available in our physiology department. Respiratory rate was counted by counting chest rises in 1 minute. To check oxygen saturation and pulse, we used a pulse oximeter by placing it on the index finger and recording the reading when there was no change in reading for 5 seconds. Before recording these parameters, all subjects were advised to relax for 5 minutes and to sit comfortably with full support. Assessment of lung function and stages of COPD was confirmed by performing standardized spirometry in both groups using a Spiro-tech Clarity model (No. ST-TGSA-0026), a portable spirometer designed in conjunction with a computer (Figure 1). Subjects were advised to relax for 5 minutes before the test began and to sit comfortably with their back supported. Subjects’ backs were kept toward the instrument so they could not see the machine ^(19)^. We record FEV1, FVC, and the ratio of FEV1/FVC in both groups after giving proper instructions and demonstration. We took three readings of all indices, and the best one was saved as data. A FEV1 of <80% of the predicted value, along with a ratio of FEV1/FVC of no more than 0.7, is considered for the diagnosis of COPD. Patients are selected according to the GOLD criteria. Patients with GOLD stages 1 and 2 were included in the study.

**Figure 2.**
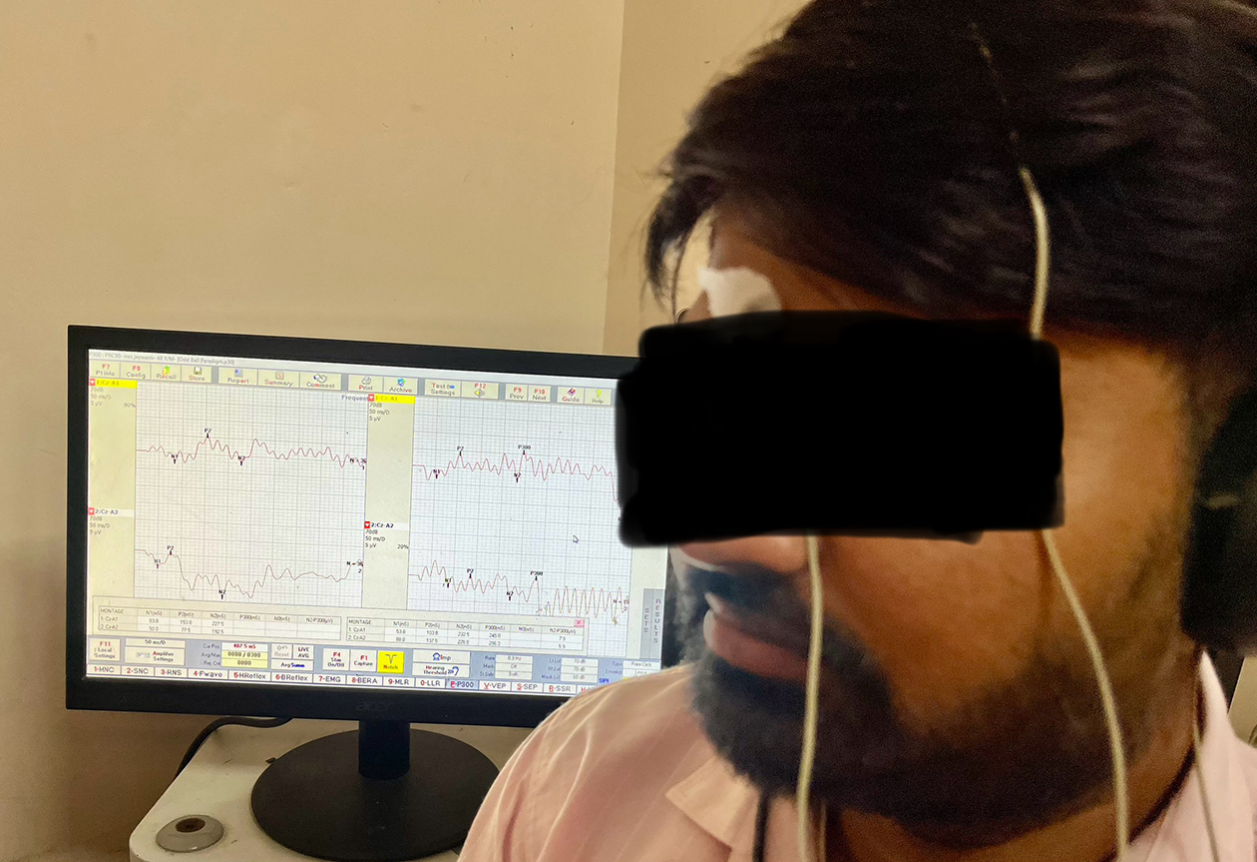
Event-related potential in neurophysiology lab.

Assessment of cognitive functions in both groups was conducted using MMSE and a machine-based objective test, the P300. MMSE questions were completed according to the patient’s preferred language, either English or Hindi. MMSE is a global assessment tool of many domains, including orientation, attention, calculation, and recall; according to the MMSE score, all subjects are grouped into three categories of CI (mild, moderate, and severe). The score was given out of a maximum score of 30. MMSE score of ≥24 is considered as normal, 19-23 as mild, 10-18 as moderate, and 9 or <9 severe as CI. We employed a typical machine-based objective procedure, the “oddball” paradigm, in which a target stimulus is presented among more frequent standard background stimuli (Figure 2). In this study, the test procedure was conducted in a silent acoustic environment at the Department of Physiology, Neurophysiology lab. All participants were instructed to sit comfortably, close their eyes, and advised not to sleep during the test procedure. The scalp was properly cleaned to reduce resistance. Then, electrodes are placed with the help of conducting paste, which increases conductivity. Silver/silver chloride (Ag-AgCl) disc electrodes were used for recording. A standard reference electrode will be placed on the Cz position of the scalp. Two active electrodes were placed on each mastoid process (A1 and A2). A ground electrode was placed on the Fpz position. Subjects were asked to wear headphones, through which they heard the rare (target) and frequent sounds (non-target) of different loudness or pitch in a ratio of 1:4. A total of 300 stimuli, delivered at a frequency of 1 stimulus per second, were presented. Subjects were instructed to recognize the rarer type of sound and raise their finger with the dominant hand each time they heard it. Recorded signals were filtered, and their latency and amplitude were used for interpretation after amplification. Auditory ERPs were recorded under standard settings, ensuring that environmental factors influencing ERPs, such as noise, temperature, or intense luminosity, were controlled during each recording ^(20)^.

Collected data were entered in Microsoft Excel software in two groups (healthy volunteers as group B and patients with COPD as group A) and analyzed using SPSS 21 software. Continuous data is expressed as mean ± standard deviation (SD), and count data is described as a proportion. Continuous data was analyzed using paired (inter-group) and unpaired (intra-group) t-tests. Correlation coefficients were calculated to find the correlation. Individuals with COPD who exhibited an increase in latency of the P300 wave or a decrease in amplitude of the P300 wave beyond ± 3 SD of healthy volunteers (HVs) were considered to have significant abnormalities. Values of P300 latency and amplitude, and MMSE score, also correlated with spirometric indices, age, sex, BMI, and other factors. The data obtained was statistically analyzed using Pearson’s correlation.

## Results

GOLD stage 1 and 2 patients with COPD in the present study were grouped as group A. The same age group of healthy subjects was used as group B. Both male and female age groups, 30 to 50 years, were included in our study. The duration of COPD, ≥5 years, was also noted in the case group. Table 1 depicts the mean age, gender distribution, residence, BMI, and socioeconomic status. Mean ± SD for age in group A was 45.95 ± 4.135 years, and in group B it was 38.65 ± 6.87 years. The groups consisted of 60% men and 40% women in group A (n = 60), and 58.3% men and 41.7% women in group B (n = 60). In group A (n = 60), 33.3% of the subjects were rural residents and 66.7% were urban residents. In group B (n = 60), 15% of the subjects were from rural areas, and 85% had a residence in a metropolitan area. The p-value was 0.0002 for socioeconomic status. A total of 38 subjects out of 60 in the study group were found to have a history of smoking, with a mean of 15.86 ± 4.12 packs per year of smoking. In the control group, no history of smoking was reported. Mean BMI in group A was noted as 22.40 with a SD of ±2.87, and in group B, it was mentioned as 24.19 ± 2.72. The result also shows a significant difference in education between groups A and B, with a p-value of 0.000082. The following variables were recorded in environmental history results showed in group A: 66.66% passive smokers, 63.33% lived in cemented houses, 65% had indoor kitchen type, 63.33% had a cooking duration of more than 15 years, 46.66% family history of COPD, 56.66% childhood respiratory infections, and 75% exposed to dust and chemicals (Table 1).

**Table 1.** Distribution of Sociodemographic Variables among Study Population.

Variables	Distribution	Group A, mean ± SD	Group B, mean ± SD	t value/X^2^ value	p Value
No of subjects	(n)	60	60		
Mean Age (years)		45.95 ± 4.135	38.65 ± 6.87	-7.052 (t value)	<0.0001
Sex % (n = 60)	Male	60 (36%)	58.3 (35%)	0.0342	0.8533
	Female	40 (24%)	41.7 (25%)		
Residence % (n = 60)	Rural	33.3 (20%)	5 (9%)	5.456	0.01
	Urban	66.7 (40%)	85 (51%)		
BMI (kg/m^2^)		22.40 ± 2.87	24.19 ± 2.72	3.507 (t-value)	0.0006
Education	Illiterate	27	12	23.9494 (X^2^ value)	0.000082
	Primary school	07	04		
	Middle school	18	13		
	Higher school	07	14		
	Graduate+ Post graduate	01	17		
SES	Lower Middle	33	14	18.0552 (X^2^ value)	0.0002
	Upper Lower	26	34		
	Upper Middle	01	12		
Smoking history	Current	13 (21.66%)	nil	---	-----
	Ex	43 (71.66%)	nil		
	Never	4 (6.66%)	nil		
	Smoking pack years	15.86 ± 4.12	nil		
Passive smoking	yes	40 (66.66%)	29 (48.33%)		
	no	20 (33.33%)	31 (51.66%)		
House type	Pucca/semipucca/ Cemented house	38 (63.33%)	40 (66.66%)		
	Kutcha	22 (36.66%)	20 (33.33%)		
Kitchen type	Outdoor	21 (35%)	19 (31.66%)		
	Indoor	39 (65%)	41 (68.33%)		
Cooking duration	<15 years	22 (36.66%)	23 (38.33%)		
	>15 years	38 (63.33%)	37 ( 61.66%)		
	<2 hours /day	28 (46.66%)	24 (40%)		
	>2 hours/day	32 (53.33%)	36 (60%)		
Working hours	Outdoor	26 (43.33%)	22 (36.66%)		
	Indoor	34 (56.66%)	38 (63.33%)		
Familial history for COPD		28 (46.66%)	-----		
Childhood respiratory infections		34 (56.66%)	32 (53.33%)		
Work exposure to dusts/chemicals		45 (75%)	24 (40%)		

BMI: body mass index; COPD: chronic obstructive pulmonary disease; SD: standard deviation; SES: Socioeconomic status

Table 2 depicts comparison of vital parameters among groups A and B. A Significant difference in saturation of both groups with p-value <0.0001.

**Table 2.** Comparison of Vital Parameters among A and B Group.

Vitals parameters	Group A (mean ± SD)	Group B (mean ± SD)	t Value	p Value
Blood pressure (mm of hg)	SBP -124.533 ± 4.63	SBP-122.63 ± 4.52	-2.275	0.0247
	DBP -80.5 ± 3.784	DBP-80.16 ± 3.02	-0.544	0.5872
Pulse (rate/min)	75.28 ± 5.266	75.63 ± 4.36	0.397	0.6924
Respiratory rate (rate/min)	14.63 ± 1.352	13.7 ± 1.96	0.397	0.0030
Saturation (%)	96.033 ± 1.50	98.13 ± 0.75	9.686	<0.0001

Unpaired t-test, χ^2^ test, p < 0.05; significant, p < 0.001; highly significant. DBP: diastolic blood pressure; SBP: systolic blood pressure; SD: standard deviation.

Table 3 shows lung function parameters in both groups. The mean FEV1 /FVC ratio was 66.69 ± 2.62 in group A and 82.63 ± 4.17 in group B with a significant p-value <0.0001.

**Table 3.** Comparison of Lung Function Parameters among A and B Groups.

Spirometric indices	Group A (mean ± SD)	Group B (mean ± SD)	t Value	p Value
FEV1 (liters/sec)	1.61 ± 2.07	2.29 ± 0.54	2.462	0.0153
FVC ( liters/sec)	2.424 ± 0.539	2.75 ± 0.56	3.249	0.0015
FEV1/FVC ratio (%)	66.69 ± 2.62	82.63 ± 4.17	25.071	<0.0001

Unpaired t-test, χ^2^ test, p < 0.05; significant, p < 0.001; highly significant. FEV1: forced expiratory volume in 1 second; FVC: forced vital capacity; SD: standard deviation.

Table 4 is about comparisons of cognitive parameters in both groups. The mean MMSE score in group A was 24.65 ± 2.05 compared to group B’s 27.56 ± 2.15, with a p-value <0.0001, showing highly significant results. In group A, 26/60 was detected with mild CI in comparison to 3/60 in control. In our study, the mean latency increased and decreased mean amplitude of the P300 wave for rare tone stimuli noted in group A as compared to group B. 19 out of 60 group A subjects were found to have a significant latency below 3SD of group B. The mean latency of group A was noted at 298.48 ± 21.98 milliseconds as compared to group B, in which the mean was reported at 265.89 ms with an SD of ± 15.80 ms with a highly significant p-value (<0.0001). Mean amplitude was found low (4.25 ± 1.502μv) in group A which was found high in group B (6.06 ± 1.94μv) showing a highly significant results (p < 0.0001) but no one group A subject were found amplitude <3SD of group B.

**Table 4.** Comparisons of Cognitive Parameters in A and B Groups.

Cognitive parameters	Group A	Group B	t Value	p Value
MMSE score	24.65 ± 2.05	27.56 ± 2.15	7.588	<0.0001
P300 Latency (milliseconds)	298.48 ± 21.98	265.89 ± 15.80	9.326	<0.0001
P300Amplitude (μv)	4.25 ± 1.502	6.06 ± 1.94	5.714	<0.0001

Unpaired t-test, χ^2^ test, p < 0.05; significant, p < 0.001; highly significant. MMSE: Mini-Mental State Examination.

## Discussion

In this study, patients with stable COPD of GOLD stage 1 and 2 with no hypoxemia were recruited, and cognitive functions were assessed by objective (P300/ERP) and subjective (MMSE) tests. The result of this study depicts that the mean MMSE score was reduced in cases compared to controls. In this study, 42% cases had CI and only 5% controls had CI on the MMSE scale. Results were similar to the study by Samareh et al. ^(21)^. In this study, results of ERP show that the mean latency was increased and mean amplitude was decreased in cases as compared to controls. In cases, 33% were found to have a significant latency <3 SD of controls, but no cases were found with an amplitude <3 SD of controls. A study done by Gupta et al. ^(22)^ reported that patients with COPD had impaired cognitive functions, which were assessed by P300, result was similar to this study. In another study, a similar finding to our study was noted by Krishnamurthy et al. ^(23)^, where the amplitude of the P300 wave obtained was also significantly reduced compared to the normal individuals. A study by Liesker et al. ^(24)^ reported lower cognitive score in patients with COPD than healthy volunteers on cognitive assessment tools other than MMSE. In the present study, cognitive function was assessed by using MMSE and P300 and results was similar to a study by Qian et al. ^(25)^ who used MoCA score to assess cognitive functions. The Kirkil et al. ^(26)^ study shows that cognitive performance evaluated by the P300 test is impaired in patients with COPD compared to healthy control. Study in contrary to this study was by Salik et al. ^(27)^ which depicts similar cognitive functions in non- hypoxemic patients with COPD as observed for healthy subjects by using MMSE score.

In the present study, cognitive functions were decrease in patients with COPD―due to chronic and progressive airway obstruction present in COPD―enhancing the adverse hypoxic effect on the brain to the extent that it manifests in impaired cognitive functions. Patients with COPD may experience CI due to a number of contributing factors, such as age, the severity and duration of the disease, hypercapnia, smoking, and vascular dysfunction. These variables cause aberrant brain structure and function, which manifests as cognitive dysfunction symptoms in people with COPD ^(28)^. A study reported that patients with COPD had a decrease in cerebral blood flow, especially in the frontal area, which causes a decrease in verbal memory in all patients with COPD as compared to control ^(29)^. Another study done by Dodd et al. ^(30)^ reported that in non-hypoxemic COPD patients, there is reduced white matter integrity throughout the brain and widespread disturbance in the functional activation of gray matter which might contribute to cognitive dysfunction. Besides lower oxygen and/ or higher carbon dioxide levels in the blood, a complex interaction between pulmonary and non-pulmonary risk factors may account for COPD-related cognitive deficits. Other major risk factors that may potentially be associated with CI are the presence of increased inflammation and oxidative stress; reduced physical activity; peripheral vascular disease, high or low blood pressure (non-normotensive patients); increased intracranial pressure associated with the narrowing of blood vessels in the brain; coexisting comorbidities; tobacco smoking; and genetic predisposition. This study explores cognitive dysfunctions in stable patients with non-hypoxemic COPD that will be helpful to future researchers to find out the cause of CI other than hypoxemia in stable non-hypoxemic patients.

The limitation of the present study was its small sample size and lack of a multicentric study. We did not correlate data with smoking history or duration of COPD. We took patients who have a history of COPD for 5 years or >5 years. To overcome the relative confounding factors of MMSE, we used ERP (P300), an objective test that is not affected by subjective influencers. However, its long range of latency and amplitude makes it challenging to interpret.

### Conclusions

Several common signs characterize CI and seem to have detrimental effects on many aspects of patient function, health status, and quality of life. They are also related to lower adherence to medical treatment and increased rates of hospitalization and mortality in COPD. The CI not only affects the physical function and health status but also aggravates mortality and disability in patients with COPD. Studies have found that the mortality rate of elderly patients with COPD with CI is nearly three times higher than that of elderly patients with CI or COPD. This phenomenon brings a substantial economic burden on the patient’s family and society. Early identification of a minimal decrease in cognition is necessary to reduce the risk of severe CI and functional decline in patients with COPD.

## Article Information

### Acknowledgments

We would like to thank all the participants who have given consent to participate in this study, the faculty of Department of Physiology, and all hospital staff.

### Author Contributions

Conceptualization: Pratima Agrawal, Neha Saboo. Study Design: Pratima Agrawal. Definition of intellectual content: Pratima Agrawal, Sudhanshu Kacker. Literature Search: Pratima Agrawal, Sudhanshu Kacker, Neha Saboo. Data Acquisition: Pratima Agrawal, Neha Saboo. Data Analysis: Pratima Agrawal, Neha Saboo. Manuscript Preparation: Pratima Agrawal, Neha Saboo. Manuscript Editing: Neha Saboo, Pratima Agrawal.

All authors read and approved the final manuscript.

### Conflicts of Interest

None

### Consent to Participate

All the participants who were willing to participate have given the consent to publish their data.

### Patient Consent Form

Written informed consent form were taken from all the participants before collecting data.

### Ethical Approval and/or Institutional Review Board (IRB) Approval

The study was approved by the institutional Ethics Committee of RUHS College of Medical Sciences, Jaipur, Rajasthan, letter no. RUHS-CMS/Ethics/Comm./2023/281 dated 31/01/2024.

## References

[1] Barnes PJ, Celli BR. Systemic manifestations and comorbidities of COPD. Eur Respir J. 2009;33(5):1165-85. 19407051 10.1183/09031936.00128008

[2] Cohen AJ, Brauer M, Burnett R, et al. Estimates and 25-year trends of the global burden of disease attributable to ambient air pollution: an analysis of data from the Global Burden of Diseases Study 2015 [published corrections in Lancet. 2017;389(10087):e15] [published corrections in Lancet. 2018;391(10130):1576]. Lancet. 2017;389(10082):1907-18. 28408086 10.1016/S0140-6736(17)30505-6 PMC5439030

[3] Verma A, Gudi N, Yadav UN, et al. Prevalence of COPD among population above 30 years in India: a systematic review and meta-analysis. J Glob Health. 2021;11:04038. 34484706 10.7189/jogh.11.04038 PMC8397327

[4] Deﬁnition of cognition in English [Internet]. Oxford Learner’s Dictionaries. 2017 [cited 2017 Mar 14]. Available from: https://www.oxfordlearnersdictionaries.com/definition/english/cognition

[5] Cleutjens FAHM, Janssen DJA, Ponds RWHM, et al. COgnitive-pulmonary disease. BioMed Res Int. 2014;2014:697825. 24738069 10.1155/2014/697825 PMC3971492

[6] Sachdev PS, Blacker D, Blazer DG, et al. Classifying neurocognitive disorders: the DSM-5 approach. Nat Rev Neurol. 2014;10(11):634-42. 25266297 10.1038/nrneurol.2014.181

[7] 2020 Global strategy for prevention, diagnosis, and management of COPD [Internet]. Global Initiative for Chronic Obstructive Lung Disease. 2021 [cited 2022 May 17]. Available from: https://goldcopd.org/archived-reports/#

[8] Pelgrim CE, Peterson JD, Gosker HR, et al. Psychological co-morbidities in COPD: targeting systemic inflammation, a benefit for both? Eur J Pharmacol. 2019;842:99-110. 30336140 10.1016/j.ejphar.2018.10.001

[9] Villeneuve S, Pepin V, Rahayel S, et al. Mild cognitive impairment in moderate to severe COPD: a preliminary study. Chest. 2012;142(6):1516-23. 23364388 10.1378/chest.11-3035

[10] Hung WW, Wisnivesky JP, Siu AL, et al. Cognitive decline among patients with chronic obstructive pulmonary disease. Am J Respir Crit Care Med. 2009;180(2):134-7. 19423714 10.1164/rccm.200902-0276OC

[11] Dodd JW, Getov SV, Jones PW. Cognitive function in COPD [published corrections in Eur Respir J. 2010;36(1):223]. Eur Respir J. 2010;35(4):913-22. 20356988 10.1183/09031936.00125109

[12] Antonelli-Incalzi R, Corsonello A, Trojano L, et al. Correlation between cognitive impairment and dependence in hypoxemic COPD. J Clin Exp Neuropsychol. 2008;30(2):141-50. 18938666 10.1080/13803390701287390

[13] Carone M, Bertolotti G, Anchisi F, et al. Analysis of factors that characterize health impairment in patients with chronic respiratory failure. Quality of life in Chronic Respiratory Failure Group. Eur Respir J. 1999;13(6):1293-300. 10445604 10.1183/09031936.99.13613019

[14] Allen SC, Jain M, Ragab S, et al. Acquisition and short-term retention of inhaler techniques require intact executive function in elderly subjects. Age Ageing. 2003;32(3):299-302. 12720616 10.1093/ageing/32.3.299

[15] Incalzi RA, Gemma A, Marra C, et al. Verbal memory impairment in COPD: its mechanisms and clinical relevance. Chest. 1997;112(6):1506-13. 9404746 10.1378/chest.112.6.1506

[16] Finney GR, Minagar A, Heilman KM. Assessment of mental status. Neurol Clin. 2016;34(1):1-16. 26613992 10.1016/j.ncl.2015.08.001

[17] Ma X, Shen J, Sun J, et al. P300 event-related potential predicts cognitive dysfunction in patients with vestibular disorders. Biomedicines. 2023;11(9):2365. 37760807 10.3390/biomedicines11092365 PMC10525252

[18] Andrianopoulos V, Gloeckl R, Vogiatzis I, et al. Cognitive impairment in COPD: should cognitive evaluation be part of respiratory assessment? Breathe (Sheff). 2017;13(1):e1-9. 29184593 10.1183/20734735.001417 PMC5702891

[19] Moore VC. Spirometry: step by step. Breathe (Sheff). 2012;8:232-40. 10.1183/20734735.5217-2011 PMC9973498 36867111

[20] Lee HJ, Kim L, Kim YK, et al. Auditory event-related potentials and psychological changes during sleep deprivation. Neuropsychobiology. 2004;50(1):1-5. 15179012 10.1159/000077933

[21] Samareh Fekri M, Hashemi-Bajgani SM, Naghibzadeh-Tahami A, et al. Cognitive impairment among patients with chronic obstructive pulmonary disease compared to normal individuals. Tanaffos. 2017;16(1):34-9. 28638422 PMC5473380

[22] Gupta PP, Sood S, Atreja A, et al. A comparison of cognitive functions in non-hypoxemic chronic obstructive pulmonary disease (COPD) patients and age-matched healthy volunteers using Mini-Mental State Examination questionnaire and event-related potential, P300 analysis. Lung India. 2013;30(1):5-11. 23661909 10.4103/0970-2113.106119 PMC3644834

[23] Krishnamurthy S, Sivagnaname Y, Gumallapu GC. Identification of subclinical cognitive impairment in chronic obstructive pulmonary disease using auditory P300 event related potential. Monaldi Arch Chest Dis. 2019;89(2):10.4081/monaldi.2019.1039. 10.4081/monaldi.2019.1039 31148604

[24] Liesker JJW, Postma DS, Beukema RJ, et al. Cognitive performance in patients with COPD. Respir Med. 2004;98(4):351-6. 15072176 10.1016/j.rmed.2003.11.004

[25] Qian H, Lin H, Li Y. Assessment of cognition and associated factors in patients with stable chronic obstructive pulmonary disease. Zhonghua Jie He He Hu Xi Za Zhi. 2014;37(10):769-73. Chinese. 25537414

[26] Kirkil G, Tug T, Ozel E, et al. The evaluation of cognitive functions with P300 test for chronic obstructive pulmonary disease patients in attack and stable period. Clin Neurol Neurosurg. 2007;109(7):553-60. 17532116 10.1016/j.clineuro.2007.03.013

[27] Salik Y, Ozalevli S, Cimrin AH. Cognitive function and its effects on the quality of life status in the patients with chronic obstructive pulmonary disease (COPD). Arch Gerontol Geriatr. 2007;45(3):273-80. 17343931 10.1016/j.archger.2006.12.002

[28] Wang T, Mao L, Wang J, et al. Influencing factors and exercise intervention of cognitive impairment in elderly patients with chronic obstructive pulmonary disease. Clin Interv Aging. 2020;15:557-66. 32368022 10.2147/CIA.S245147 PMC7183549

[29] Ortapamuk H, Naldoken S. Brain perfusion abnormalities in chronic obstructive pulmonary disease: comparison with cognitive impairment. Ann Nucl Med. 2006;20(2):99-106. 16615418 10.1007/BF02985621

[30] Dodd JW, Chung AW, van den Broek MD, et al. Brain structure and function in chronic obstructive pulmonary disease: a multimodal cranial magnetic resonance imaging study. Am J Respir Crit Care Med. 2012;186(3):240-5. 22652026 10.1164/rccm.201202-0355OC

